# Tunable Optical Bistability in Asymmetric Dielectric Sandwich with Graphene

**DOI:** 10.3390/nano16020116

**Published:** 2026-01-15

**Authors:** Qiawu Lin, Wenyao Liang, Renlong Zhou, Sa Yang, Shuang Li

**Affiliations:** 1 School of Physics and Information Engineering, Guangdong University of Education, Guangzhou 510303, China; linqiawu@gdei.edu.cn (Q.L.); yangsa@gdei.edu.cn (S.Y.); lishuang808@gdei.edu.cn (S.L.); 2 School of Physics, South Normal University, Guangzhou 510006, China; 3 School of Physics and Optoelectronics, South China University of Technology, Guangzhou 510640, China

**Keywords:** optical bistability, asymmetric dielectric sandwich, graphene

## Abstract

This study theoretically investigates the nonlinear optical response of asymmetric dielectric structures embedded with graphene and demonstrates tunable optical bistability in the terahertz frequency range. Our findings reveal that the bistable behavior can be effectively modulated by varying the incident angle, the working wavelength, and the thickness and permittivity of the dielectric layers. In symmetric dielectric configurations, transmittance is enhanced, whereas in asymmetric structures, it is reduced. The thresholds of optical bistability decrease with increasing wavelength of the incident light, while they increase with thicker dielectric layers or higher permittivity of the dielectric medium. Furthermore, widening the bistability range can be achieved by increasing the incident angle. The proposed asymmetric graphene–dielectric layered structure offers a promising platform for the development of advanced terahertz active photonic devices, including optical modulators, optical switches, and mid-infrared functional components.

## 1. Introduction

Optical bistability (OB) refers to the existence of two stable output light intensity states for a single input intensity, which can be switched between each other. The input-output characteristic typically exhibits an S-shaped hysteresis loop. Consequently, one input level corresponds to two possible stable output states, with the actual output value depending on the historical trend of the input variation [1]. This phenomenon originates from the nonlinear interaction between light and matter. Under strong incident electromagnetic fields, the induced electric dipole oscillations in dielectric atoms become nonlinear with respect to the external field, generating polarization fields and secondary radiation that lead to this specific nonlinear optical effect, often regarded as a feedback mechanism in nonlinear dielectric systems [2].

Regarding the bistability effect, the pursuit of higher photoswitching speeds and control efficiency drives the development of miniaturized device structures. Various novel configurations have been explored, including photonic crystal cavities [3], subwavelength metal gratings [4], metal gap waveguide nanocavities [5], nano-antenna arrays composed of nonlinear materials [6,7], and metamaterials [8]. However, achieving significant nonlinear effects in conventional Kerr materials (e.g., Si, GaAs) often requires substantial interaction lengths, which conflicts with the miniaturization demands for integrated optical devices. This limitation has motivated the search for alternative materials possessing strong inherent nonlinearities.

While traditional and emerging nonlinear materials (e.g., topological insulators, transition metal dichalcogenides) exhibit third-order nonlinearity, graphene stands out for its exceptionally high nonlinear susceptibility [9,10], ultrafast optical response, and gate-tunable conductivity [11,12]. Moreover, its atomic-scale thickness also makes it ideal for compact, tunable, and low-threshold optical devices without significant optical path length. Leveraging its excellent nonlinear optical characteristics, graphene has been successfully employed to realize optical modulators [13,14]. Furthermore, its high-speed optical signal processing capability enables applications in optical transistors [15], all-optical switches [16], and optical memories [17]. By exploiting its tunable conductivity, graphene has also been integrated into devices such as tuned optical sensors [18], metamaterials [19,20], terahertz absorbers [21], and terahertz radiation sources [22,23,24]. Additionally, the third-order nonlinearity of graphene facilitates the fabrication of mode-locked lasers [25] and optical limiters [26]. Recent research has demonstrated OB in graphene-Kerr nonlinear substrate configurations [27], experimentally verified ultrafast all-optical switching in graphene nanomaterials [28], observed OB for monolayer graphene in an air-enclosed cavity [29], and investigated bistable phenomena in symmetric double-layer dielectric structures incorporating nonlinear graphene sheets [30]. Furthermore, a mid-infrared hyperbolic microcavity with tunable resonance was realized based on a non-periodic graphene-dielectric stack, where its optical response and the topological transition between elliptic and hyperbolic dispersion were actively controlled via gate voltage [31]. In the terahertz regime, a reconfigurable logic device was demonstrated using a metamaterial composed of patterned graphene circular rings, where frequency-multiplexed logic gates (OR, XNOR, NAND) were implemented and tuned by electrically modulating the graphene’s Fermi level [32]. However, the optical bistable behavior of graphene in asymmetric multilayer dielectric structures, particularly the influence of structural asymmetry on the bistability threshold and tuning characteristics, still lacks systematic investigation.

In this work, we investigate the optical bistable phenomena in asymmetric multilayer dielectric structures that incorporate nonlinear graphene sheets. We systematically examine the influence of asymmetric conditions on OB, including variations in dielectric permittivity, layer thickness, incident angle, and the wavelength of the incident light. This study aims to provide insights beneficial for advancing the application of nonlinear optical elements in optical information processing.

## 2. Theoretical Models and Methods

**Figure 1:**
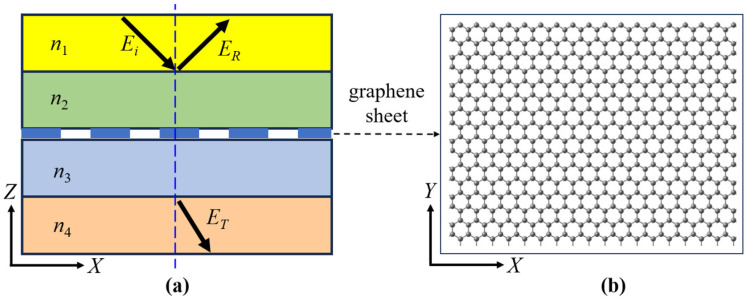
The structure of multilayer dielectric medium (**a**) containing graphene sheets (**b**). *E_i_*, *E_R_*, and *E_T_* are the amplitudes of the incident, reflected and transmitted lights.

We consider the transmission of light in a multilayer dielectric medium containing graphene sheets. The structure is shown in Figure 1a, together with the embedded graphene sheet shown in Figure 1b. It is a symmetric structure when *n*_1_ = *n*_4_ and *n*_2_ = *n*_3_, otherwise it is an asymmetric structure.

Without considering the effect of the external magnetic field, the conductivity of the graphene sheet in the dielectric medium can be described by the Kubo formula [9,27],

(1) $$\begin{array}{l} \sigma =\sigma_{0}+\sigma^{\prime }=\frac{ie^{2}k_{B}T}{\pi \hbar^{2}(\omega +\tau^{-1}i)}[\frac{E_{F}}{k_{B}T}+2\ln(e^{-\frac{E_{F}}{k_{B}T}}+1)]\\ +\frac{ie^{2}}{4\pi \hbar }\ln\left|\frac{2E_{F}-(\omega +\tau^{-1}i)\hbar }{2E_{F}+(\omega +\tau^{-1}i)\hbar }\right|-i\frac{3e^{2}{\left(ev_{F}\right)}^{2}}{32\pi \hbar^{2}E_{F}\omega^{3}}(1+\alpha_{T})\; \end{array}$$

where $\sigma_{0}$ is the conductivity without the nonlinear effect of graphene sheet, as shown below,

(2) $$\sigma_{0}=\frac{ie^{2}k_{B}T}{\pi \hbar^{2}(\omega +\tau^{-1}i)}[\frac{E_{F}}{k_{B}T}+2\ln(e^{-\frac{E_{F}}{k_{B}T}}+1)]+\frac{ie^{2}}{4\pi \hbar }\ln\left|\frac{2E_{F}-(\omega +\tau^{-1}i)\hbar }{2E_{F}+(\omega +\tau^{-1}i)\hbar }\right|$$

and $\sigma^{\prime }$ is the conductivity introduced when taking into account the nonlinear effect of the graphene sheet, as shown as below,

(3) $$\sigma^{\prime }=-i\frac{3e^{2}{\left(ev_{F}\right)}^{2}}{32\pi \hbar^{2}E_{F}\omega^{3}}(1+\alpha_{T})$$

where $\alpha_{T}$ is a two-photon absorption coefficient, ω is the angular frequency of the incident light, *e* is the electron charge, $k_{B}$ is the Boltzmann constant, *T* is the temperature, ℏ is the reduced Planck constant, τ is the relaxation time of phonon-electron interaction, $E_{F}$ is the Fermi level of graphene, and $v_{F}$ is the Fermi velocity of the electrons, $v_{F}=10^{6}\;m/s$.

The coordinate system is selected as shown in Figure 1. We study the TE mode electromagnetic wave in dielectric medium, and the propagation equation in media layer 1 is expressed as

(4) $$\begin{array}{l} E_{1y}=E_{i}\exp\left\{i*[k_{1z}(z+d_{2})+k_{x}x]\right\}+E_{R}\exp\left\{-i*[k_{1z}(z+d_{2})-k_{x}x]\right\}\\ H_{1x}=-\frac{k_{1z}}{\mu_{o}\omega }E_{i}\exp\left\{i*[k_{1z}(z+d_{2})+k_{x}x]\right\}+\frac{k_{1z}}{\mu_{o}\omega }E_{R}\exp\left\{-i*[k_{1z}(z+d_{2})-k_{x}x]\right\}\\ H_{1z}=-\frac{k_{x}}{\mu_{o}\omega }E_{i}\exp\left\{i*[k_{1z}(z+d_{2})+k_{x}x]\right\}+\frac{k_{x}}{\mu_{o}\omega }E_{R}\exp\left\{-i*[k_{1z}(z+d_{2})-k_{x}x]\right\} \end{array}$$

For the electromagnetic wave through the layer 1 dielectric medium to the layer 2 dielectric medium, the propagation equation in the layer 2 is expressed as,

(5) $$\begin{array}{l} E_{2y}=A\exp[i*(k_{2z}z+k_{x}x)]+B\exp[-i*(k_{2z}z-k_{x}x)]\\ H_{2x}=-\frac{k_{2z}}{\mu_{o}\omega }A\exp[i*(k_{2z}z+k_{x}x)]+\frac{k_{2z}}{\mu_{o}\omega }B\exp[-i*(k_{2z}z-k_{x}x)]\\ H_{2z}=-\frac{k_{x}}{\mu_{o}\omega }A\exp[i*(k_{2z}z+k_{x}x)]+\frac{k_{x}}{\mu_{o}\omega }B\exp[-i*(k_{2z}z-k_{x}x)] \end{array}$$

The propagation equation in the layer 3 dielectric medium is given by,

(6) $$\begin{array}{l} E_{3y}=C\exp[i*(k_{3z}z+k_{x}x)]+D\exp[-i*(k_{3z}z-k_{x}x)]\\ H_{3x}=-\frac{k_{3z}}{\mu_{o}\omega }A\exp[i*(k_{3z}z+k_{x}x)]+\frac{k_{3z}}{\mu_{o}\omega }D\exp[-i*(k_{3z}z-k_{x}x)]\\ H_{3z}=-\frac{k_{x}}{\mu_{o}\omega }C\exp[i*(k_{3z}z+k_{x}x)]+\frac{k_{x}}{\mu_{o}\omega }D\exp[-i*(k_{3z}z-k_{x}x)] \end{array}$$

In layer 4,

(7) $$\begin{array}{l} E_{4y}=E_{T}\exp\left\{i*[k_{4z}(z-d_{3})+k_{x}x]\right\}\\ H_{4x}=-\frac{k_{4z}}{\mu_{o}\omega }E_{T}\exp\left\{-i*[k_{4z}(z-d_{3})+k_{x}x]\right\}\\ H_{4z}=-\frac{k_{x}}{\mu_{o}\omega }E_{T}\exp\left\{i*[k_{4z}(z-d_{3})+k_{x}x]\right\} \end{array}$$

$k_{x}=k_{o}\sqrt{\varepsilon_{1}}\sin\theta$, $k_{yz}=\sqrt{k_{0}^{2}\varepsilon_{j}-{k_{x}}^{2}},j=1,2,3,4$, and θ is the angle of incidence. According to the boundary conditions of the electromagnetic field, $Z=0,H_{2x}=H_{3x}=\sigma E_{2y}$, and we can get

(8) $$\begin{array}{l} \Pi =\frac{1}{8}\left\{(1+\frac{k_{2z}}{k_{1z}})[(1-\frac{k_{3z}}{k_{2z}})\Gamma +\Xi ]\exp(-ik_{2z}d_{2})+\right.\\ \left.\left[(1-\frac{k_{2z}}{k_{1z}})[(1+\frac{k_{3z}}{k_{2z}})\Gamma -\Xi ]\exp(ik_{2z}d_{2}\right)\right\} \end{array}$$

and

(9) $$\Gamma =(1+\frac{k_{4z}}{k_{3z}})\exp(-ik_{3z}d_{3})+(1-\frac{k_{4z}}{k_{3z}})\exp(ik_{3z}d_{3})$$

(10) $$\Xi =2\frac{k_{3z}}{k_{2z}}(1+\frac{k_{4z}}{k_{3z}})\exp(-ik_{3z}d_{3})-\frac{\mu_{0}\omega }{k_{2z}}(\sigma_{0}+\frac{1}{4}\sigma^{\prime }{\left|E_{T}\right|}^{2}{\left|\Gamma \right|}^{2})\Gamma$$

So,

(11) $${\left|E_{i}\right|}^{2}={\left|E_{T}\right|}^{2}{\left|\Pi \right|}^{2}$$

For convenience, we have added Table 1 below to summarize all material parameters used in the simulations. The table includes dielectric permittivities, layer thicknesses, graphene parameters, and incident conditions. It should be noted that in our model, the dielectric layers are treated as non-dispersive within the studied terahertz range, which is a common simplification for the theoretical analysis of bistability thresholds. However, graphene’s conductivity is frequency-dependent and is described by the Kubo formula (Equations (2) and (3)). This dispersion is essential for accurately capturing graphene’s nonlinear optical response.

## 3. Results and Discussions

The transfer matrix method (TMM) is employed to model wave propagation in the layered structure. All simulations are implemented in MATLAB software (Matlab.v7.1.R14.SP3) using a self-consistent iterative approach to handle graphene’s nonlinear conductivity. Perfectly matched layers are applied at both ends of the structure to mimic an infinite extent. It is noted that dielectric layers are assumed lossless and non-dispersive, while graphene is modeled as an infinitesimally thin sheet with surface conductivity; interfacial roughness and defects are neglected.

The third-order nonlinear effect of graphene influences the propagation of electromagnetic waves in dielectric media. Based on wave propagation theory, we simulate the transmission of electromagnetic waves through symmetric and asymmetric dielectric layers embedded with graphene sheets. The influence of graphene’s nonlinearity on wave propagation is analyzed, and tunable optical bistability is demonstrated. It is shown that such bistability can be controlled by adjusting the incident angle, as well as by varying the thickness and permittivity of the dielectric slabs.

**Figure 2:**
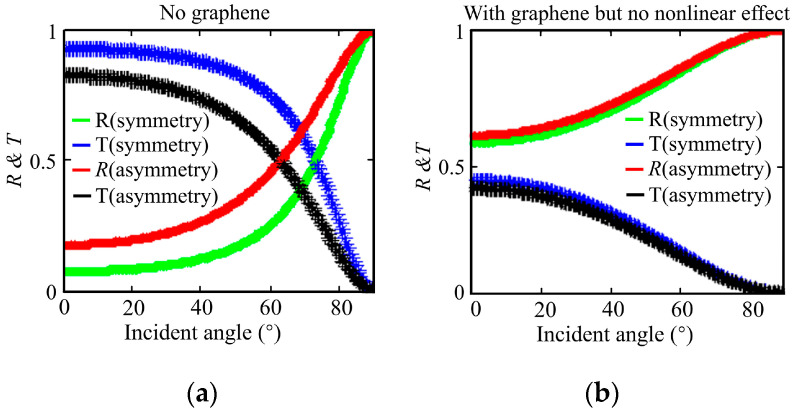
Transmittance and reflectivity versus incident angle for symmetric and asymmetric structures (**a**) without and (**b**) with graphene. Parameters for the asymmetric structure: *ε*_1_ = 1, *ε*_2_ = 2.75, *ε*_3_ = 2.25, and *ε*_4_ = 1.25. Symmetric structure: *ε*_1_ = 1, *ε*_2_ = 2.25, *ε*_3_ = 2.25, and *ε*_4_ = 1. Other parameters: wavelength *λ* = 100 μm, Fermi energy *E_F_* = 0.8 eV, *T* = 300 K, *τ*^−1^ = 0, *d*_2_ = *d*_3_ = 4 μm, and *d*_1_ = *d*_4_ are sufficiently large.

Prior to examining optical bistability, we first compare the transmission of electromagnetic waves in dielectric layers with and without graphene, disregarding its third-order nonlinear effect. Because graphene affects the interface behavior, a comparison between Figure 2a,b reveal that the presence of graphene enhances reflectivity and reduces transmittance. In Figure 2b, under the same incident angle, the asymmetric structure exhibits higher reflectivity than the symmetric one, facilitating the achievement of total internal reflection (TIR). Furthermore, when the third-order nonlinear effect is neglected, reflectivity increases with the incident angle. Additionally, adjusting the permittivity of the asymmetric dielectric layer can further enhance reflectivity.

Actually, the OB in our structure originates from the intensity-dependent third-order nonlinear conductivity of graphene. As the incident field intensity increases, the resulting nonlinear conductivity modifies the effective refractive index sensed by the propagating wave. This, in turn, shifts the resonant condition of the asymmetric Fabry–Perot-like cavity formed by the dielectric layers. Once the input intensity exceeds a critical threshold, the interaction between graphene’s nonlinear response and the cavity feedback leads to a switching behavior between distinct high- and low-transmission states, giving rise to the characteristic hysteresis.

**Figure 3:**
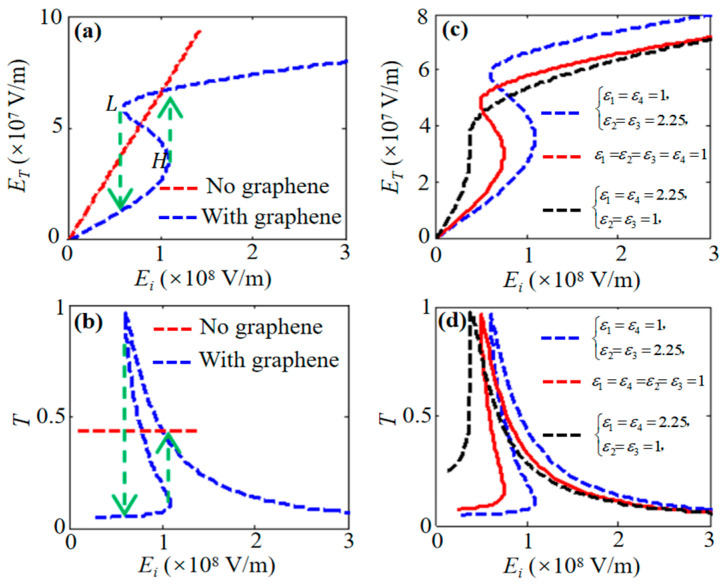
Transmitted intensity (**a**) and transmittance (**b**) versus incident intensity for symmetric structures without and with graphene nonlinearity. (**c**,**d**) Changing permittivity alters the bistability threshold. Parameters: Symmetric case (blue line): *ε*_1_ = *ε*_4_ = 1, *ε*_2_ = *ε*_3_ = 2.25. Vacuum case (red line): *ε*_1_ = *ε*_2_ = *ε*_3_ = *ε*_4_ = 1. Inverted case (black line): *ε*_1_ = *ε*_4_ = 2.25, *ε*_2_ = *ε*_3_ = 1. Incidence angle *θ* = 75°. Other parameters are as in Figure 2.

We then investigate optical bistability arising from the third-order nonlinear effect of graphene in a symmetric dielectric medium (Figure 1). In this structure, layers 1 and 4 and layers 2 and 3 are identical, forming a simple symmetric stack. Optical bistability is achieved via graphene’s nonlinearity, as shown in Figure 3a,b. In Figure 3a, the red line (without graphene nonlinearity) shows a proportional relationship between transmitted and incident intensities; consequently, the corresponding transmittance in Figure 3b is constant, exhibiting no bistability. When graphene nonlinearity is included, the blue line in Figure 3a exhibits a characteristic hysteresis “S” curve. Thus, optical bistability depends on graphene’s third-order nonlinear effect: transmissivity varies with incident intensity, where a single incident value can correspond to two possible transmission states, depending on the direction of intensity change. We further analyze the influence of layer permittivity on bistability in Figure 3c,d. A single graphene sheet in vacuum exhibits optical bistability (Figure 3c, red line). As incident intensity increases beyond a high threshold (*H*), the transmission jumps up. As intensity decreases below a low threshold (*L*), it jumps down, transitioning to a TIR mode. Figure 3c,d show that permittivity modification shifts these thresholds. Notably, if the outer layer permittivity significantly exceeds the inner layer’s, bistability can disappear.

**Figure 4:**
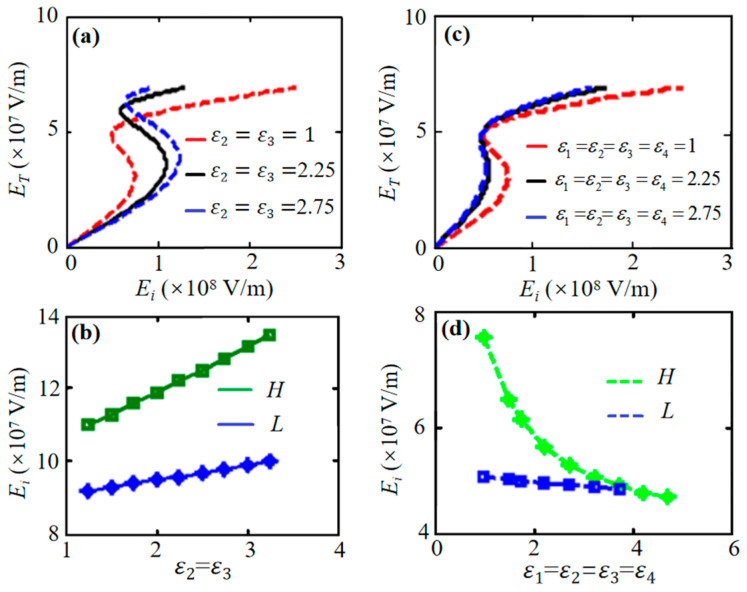
(**a**,**c**) Transmitted versus incident intensity for symmetric structures with graphene nonlinearity. (**b**) Threshold increases with increasing *ε*_2_ = *ε*_3_ (*ε*_1_ = *ε*_4_ = 1). (**d**) Threshold decreases when increasing a common permittivity of *ε*_1_ = *ε*_2_ = *ε*_3_ = *ε*_4_. Other parameters are the same as those in Figure 2.

As shown in Figure 4a,b, for a structure with *ε*_1_ = *ε*_4_ = 1 (vacuum) and *ε*_2_ = *ε*_3_, simultaneously increasing *ε*_2_ and *ε*_3_ raises the bistability threshold. Conversely, when graphene is surrounded by identical dielectric (*ε*_1_ = *ε*_2_ = *ε*_3_ = *ε*_4_), increasing this common permittivity reduces the threshold, as shown in Figure 4c,d. Excessively high permittivity of the surrounding dielectric can eliminate bistability.

**Figure 5:**
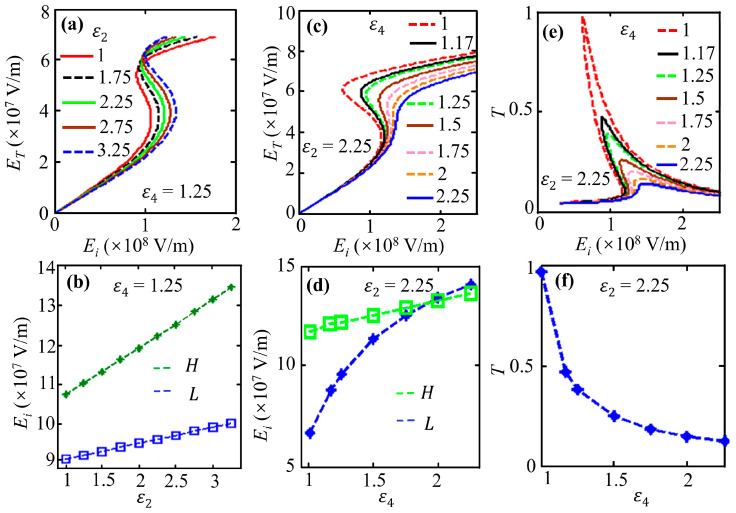
Transmitted versus incident intensity for an asymmetric structure with graphene’s third-order nonlinearity. Varying *ε*_2_ (or *ε*_4_) alters the bistability threshold (**a**–**f**). Parameters: *λ* = 100 μm, *E_F_* = 0.8 eV, *τ*^−1^ = 0, *T* = 300 K, *θ* = 75°, and *d*_2_ = *d*_3_ = 4 μm.

We further study the influence of permittivity on the bistability in an asymmetric system, as shown in Figure 5. Starting from a basic asymmetric structure (*ε*_1_ = 1, *ε*_2_ = 2.25, *ε*_3_ = 2.75, and *ε*_4_ = 1.25), increasing *ε*_2_ from 1 to 3.25 (with fixed *ε*_4_ = 1.25) raises the bistability threshold (see Figure 5a,b). Similarly, increasing *ε*_4_ from 1 to 2.25 (with fixed *ε*_2_ = 2.25) also increases the threshold (see Figure 5c,d), while reducing transmissivity (see Figure 5e,f).

**Figure 6:**
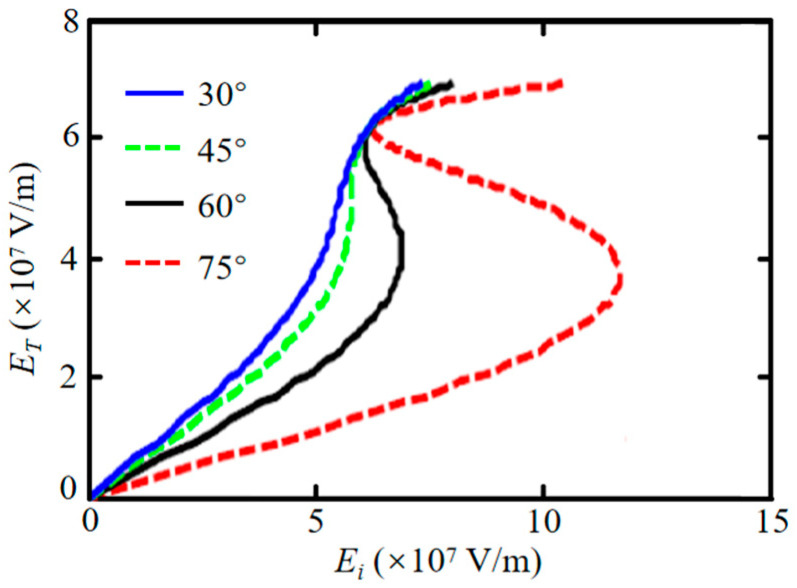
Effect of incident angle on the bistability threshold for an asymmetric structure. Other parameters: *λ* = 100 μm, *E_F_* = 0.8 eV, *τ*^−1^ = 0, *T* = 300 K, *θ* = 75°, and *d*_2_ = *d*_3_ = 4 μm.

Next, we vary the incident angle to study its influence on optical bistability. As shown in Figure 6, for an asymmetric structure (*ε*_1_ = 1, *ε*_2_ = 2.25, *ε*_3_ = 2.75, and *ε*_4_ = 1), increasing the incident angle from 30° to 75° raises the high threshold value, while the low threshold remains relatively unchanged, thereby widening the bistability range. Bistability vanishes below a certain incident angle.

**Figure 7:**
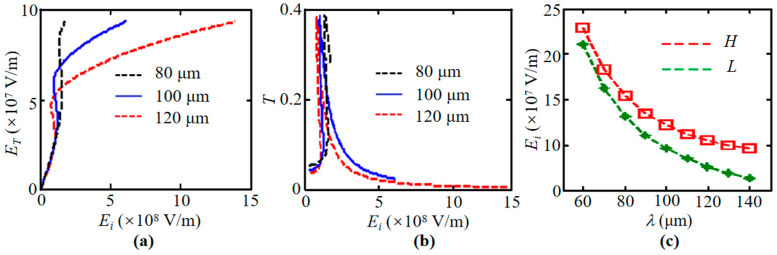
Transmitted intensity (**a**) and transmittance (**b**) versus incident intensity for the asymmetric bistable state with increasing wavelength *λ* = (80, 100, 120) μm. Other parameters are as in Figure 2. (**c**) The thresholds of optical bistability (*H* and *L*) versus *λ*.

Now, we discuss the influence of the wavelength of the incident electromagnetic wave on optical bistability. In an asymmetric structure of *ε*_1_ = 1, *ε*_2_ = 2.25, *ε*_3_ = 2.75, and *ε*_4_ = 1.25, the transmitted intensity and transmittance for *λ* = 80, 100, and 120 μm are shown in Figure 7a,b, respectively. One can find that the thresholds of optical bistability (*H* and *L*) decrease with increasing wavelength, as shown in Figure 7c. It should be noticed that the transmittance curve has a slight leftwards shift with increasing wavelength of the incident electromagnetic wave.

**Figure 8:**
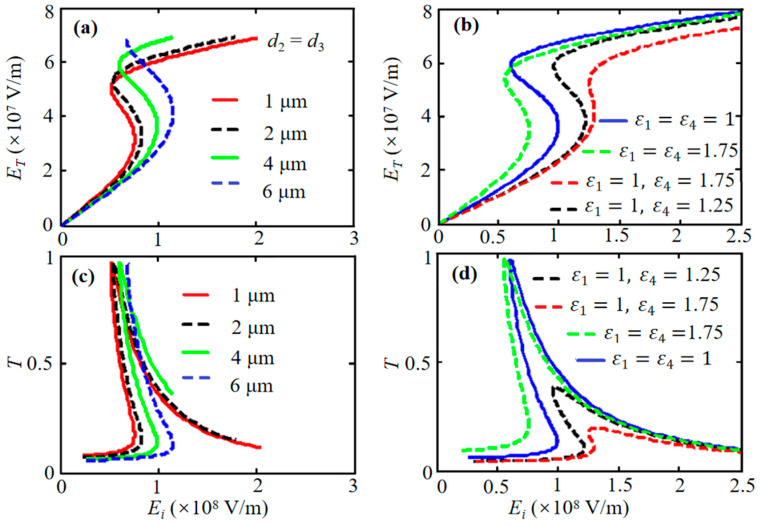
The relation of transmittance intensity and incident intensity for asymmetric structures (*ε*_1_ = 1.25, *ε*_2_ = 2.25, *ε*_3_ = 2.75, and *ε*_4_ = 1.25) with the third-order nonlinear effect of the graphene sheet. (**a**,**c**) Effect of layer thickness (*d*_2_ = *d*_3_) on bistability threshold. (**b**,**d**) Comparison of bistability for cases of *ε*_1_ = *ε*_4_ and *ε*_1_ ≠ *ε*_4_. Other parameters are the same as those in Figure 2.

Finally, we study the effect of dielectric layer thickness on optical bistability in asymmetric structures. The calculation results indicate that simultaneously varying the thickness of *d*_2_ and *d*_3_ in an asymmetric structure (*ε*_1_ = 1, *ε*_2_ = 2.25, *ε*_3_ = 2.75, and *ε*_4_ = 1.25) affects bistability: a larger thickness yields a higher threshold (Figure 8a,c). Comparison between the cases of *ε*_1_ = *ε*_4_ and *ε*_1_ ≠ *ε*_4_ (Figure 8b vs. Figure 8d) shows that matching the permittivity of the outer layers can significantly increase the bistability threshold and enhance transmittance.

Our work is distinguished from recent advances in graphene-based bistable systems—such as low-temperature superconducting multilayers [33], sandwich structures utilizing topological interface modes [34], and systems exploiting Anderson localization in random plasmonic gratings [35]. While these approaches achieve threshold modulation through external cryogenic conditions, intricate topological designs, or engineered disorder, we introduce a distinct paradigm based on intrinsic structural asymmetry in dielectric-graphene multilayers. We demonstrate that simple asymmetry in layer permittivity and thickness enables the broad tunability of bistability thresholds and hysteresis width without cryogenic conditions, complex patterning, or random gratings. This method provides a compelling alternative to realize the OB effect, thereby offering a more straightforward and potentially robust pathway to practical tunable terahertz bistable devices.

## 4. Conclusions

In summary, this study demonstrates that the third-order nonlinear optical response of graphene enables tunable optical bistability in dielectric-graphene multilayer structures. The key findings include: (1) Graphene enhances reflectivity and suppresses transmittance in linear regimes, facilitating conditions for total internal reflection. (2) Optical bistability emerges from graphene’s nonlinearity, exhibiting hysteresis dependence on incident intensity. (3) The bistability threshold can be effectively controlled by structural parameters: it increases with higher permittivity in adjacent dielectric layers (in symmetric configurations) or in specific asymmetric layer configurations, but vanishes if outer-layer permittivity becomes excessively large. (4) Increasing the incident angle widens the bistability range, while longer wavelengths lower the threshold. (5) Layer thickness and symmetry also provide control, with matched outer-layer permittivity significantly boosting transmittance. These results offer a theoretical and parametric foundation for designing graphene-based nonlinear photonic devices, such as optical switches, logic elements, and sensors.

## Figures and Tables

**Table 1:** Parameters used in simulations.

Parameters	Symbol	Value/Range
Dielectric permittivity	ε_1_, ε_2_, ε_3,_ ε_4_	1.0–3.0
Layer thicknesses	*d*_1_, *d*_2_, *d*_3_, *d*_4_	2–10 μm
Graphene Fermi energy	*E_F_*	0.8 eV
Temperature	*T*	300 K
Relaxation time	*τ*	∞, (lossless)
Incident wavelength	*λ*	80–120 μm
Incident angle	*θ*	0–90°

## Data Availability

The data presented in this study are available on request from the corresponding author.
